# Dysregulation in Plasma *ω*3 Fatty Acids Concentration and Serum Zinc in Heavy Alcohol-Drinking HCV Patients

**DOI:** 10.1155/2020/7835875

**Published:** 2020-06-09

**Authors:** Vatsalya Vatsalya, Ruchita Agrawal, Jane Frimodig, Shweta Srivastava, Melanie L. Schwandt

**Affiliations:** ^1^Department of Medicine, University of Louisville, Louisville, KY, USA; ^2^Robley Rex VA Medical Center, Louisville, KY, USA; ^3^University of Louisville, Alcohol Research Center, Louisville, KY, USA; ^4^National Institute on Alcohol Abuse and Alcoholism, NIH, Bethesda, MD, USA; ^5^Department of Psychiatry, University of Louisville, Louisville, KY, USA; ^6^Environmental Health Institute, University of Louisville, Louisville, KY, USA

## Abstract

Alcohol use disorder (AUD) patients comorbid with hepatitis C virus (HCV) infection (HCV + AUD) could have progressively severe clinical sequels of liver injury and inflammation. Serum zinc and several polyunsaturated fatty acids (PUFAs) get dysregulated in AUD as well as HCV. However, the extent of dysregulation of PUFAs and zinc deficiency and their interaction in HCV + AUD as a comorbid pathology has not been studied. We examined the role of dysregulation of FAs and low zinc in HCV + AUD patients. 138 male and female participants aged 21–67 years were grouped as HCV-only (Gr. 1; *n* = 13), HCV + AUD (Gr. 2; *n* = 25), AUD without liver injury (Gr. 3; *n* = 37), AUD with liver injury (Gr. 4; *n* = 51), and healthy volunteers (Gr. 5 or HV; *n* = 12). Drinking history, individual demographic measures, fasting fatty acids, liver function, and zinc were measured and analyzed. HCV + AUD patients showed the highest ALT level compared to the rest of the groups. Serum zinc concentrations were the lowest, and the proinflammatory shift was the highest (characterized by *ω*6 : *ω*3 ratio) in the HCV + AUD patients. Total *ω*3, eicosapentaenoic acid (EPA), and docosapentaenoic acid (DPA5,3) were the lowest in HCV + AUD patients. Total *ω*3, *α*-linoleic acid (*α*-LA) along with covariable number of drinking days past 90 days (NDD90), eicosapentaenoic acid (EPA), and docosapentaenoic acid (DPA5,3) independently showed significant association with low zinc in the HCV + AUD patients. Heavy drinking pattern showed that NDD90 has a significant mediating role in the representation of the relationship between candidate *ω*3 PUFAs and zinc uniquely in the HCV + AUD patients. Low serum zinc showed a distinctively stronger association with total and candidate *ω*3s in the HCV + AUD patients compared to the patients with HCV or AUD alone, supporting dual mechanism involved in the exacerbation of the proinflammatory response in this comorbid cohort. This trial is registered with NCT#00001673.

## 1. Introduction

Hepatitis C virus (HCV) is the most common chronic blood-borne infection, affects 5 million Americans, and causes very high morbidity and mortality [1]. Hepatitis C virus (HCV) has lasting morbidity and lasting effect on the liver and overall health [2]. Alcohol use disorder (AUD) is known to be consequential in alcoholic liver disease (ALD) [3]. ALD is a major form of liver disease that is responsible for the mortality and morbidity in the USA and worldwide [4]. With no FDA approved drug for the treatment of ALD, treatment development for the alcohol-related liver disease remains an unmet need [5]. AUD diagnosed individuals with HCV infection (HCV + AUD) have reported a propensity for liver injury compared with AUD patients, with a prevalence of HCV and AUD comorbidity being 16.3% [6].

Fatty acid synthesis has been shown to regulate Hepatitis C Virus (HCV) entry and production (Yang, 2008), though HCV has also been involved in altering fatty acid metabolism thereby causing lipid accumulation in the liver (Yamaguchi, 2005). Fatty acids are also variable with alcohol use; they can be either anti- or proinflammatory in nature [7]. The role of zinc in antiviral immunity is given in [8], and changes in zinc levels in HCV-infected patients at pre-and posttreatment evaluations were given in [9]. Recent studies have shown that zinc deficiency is commonly found in AUD, AUD with early stage of ALD, and AUD with viral infections comorbidity [9–11]. Zinc plays an important role as a cofactor in the downstream regulation of proinflammatory (*ω*6) and anti-inflammatory (*ω*3) PUFAs [12]. Studies on low zinc and *ω*3 PUFAs have been well studied separately in AUD patients with or without liver injury as well as HCV-infected patients. However, the representation and relationship of lower zinc and *ω*3 PUFAs is not clear in AUD patients who are also infected with HCV.

Hepatitis C infection and excessive alcohol exposure are synergistic in their destructive effects on the liver [13]. Identifying the early changes in the rearrangement of fatty acids, inflammation, and injury-associated nutritional measures during patient evaluation could elucidate the comorbid condition of HCV and AUD. Markers of heavy drinking [14] and profile have shown high predictability with the exacerbation of liver injury in early stage of ALD. Nonetheless, the role of such heavy drinking markers in the proinflammatory response as characterized by the dysregulation of *ω*3 PUFAs and low zinc has not been investigated in HCV-infected AUD patients. These gaps remain an open area to investigate at both the clinical level and preclinical level.

We aimed to characterize the variability in the fatty acid profile (both specific and group-based) participating in inflammation and changes in serum zinc levels in newly acquired-HCV patients with comorbid AUD diagnosis. We also evaluated the association of HCV-viral load and serum zinc and PUFAs in HCV patients with or without AUD to show the extent of linked response in this comorbid patient cohort.

## 2. Methodology

### 2.1. Patient Recruitment

The primary patient cohort of this study was AUD patients with HCV infection; other cohorts were included for comparison purposes only. This investigation was performed as one of the several projects under a larger clinical study approved by the Institutional Review Board of the National Institute on Alcohol Abuse and Alcoholism (NIAAA), National Institutes of Health (NIH), Bethesda, MD, USA (ClinicalTrials.gov identifier # NCT00001673). This trial was approved under a screening protocol 05-AA-0121 of NIAAA. All the study participants signed the informed consent prior to the involvement in the study. Data were collected and analyzed at the NIH, Bethesda, MD, USA, and University of Louisville, Louisville, KY, USA. 138 male and female participants aged 21–65 years were enrolled for this study (Table 1). The distribution of these participants was as follows: Gr. 1: HCV-only (*n* = 13); Gr. 2: HCV + AUD (*n* = 25); Gr. 3: AUD without liver injury (*n* = 37); Gr. 3: AUD with liver injury (*n* = 51); Gr. 5: healthy volunteers (HV, *n* = 12). All heavy drinkers met the diagnosis criteria of AUD according to the DSM-IV, based on the SCID I interview. Patients were not enrolled in the study if they had a diagnosis for severe psychiatric illness, suicidal, or violent tendencies or showed agitation requiring immediate clinical treatment. Patients with other forms of significant psychiatric illnesses (unless stable, and not requiring medication such as antidepressants, lithium, neuroleptics, naltrexone, acamprosate, disulfiram, benzodiazepines, or antiepileptic compounds within the last four weeks) were not enrolled. Advanced lung disease, unstable cardiovascular disease, renal failure (creatinine clearance 30 ml/min), advanced liver disease (hepatocellular carcinoma, clinically evident alcoholic hepatitis, and cirrhosis), and HIV were other exclusionary criteria. Other exclusions on the day of assessment were (1) pregnancy (negative test required) or ongoing breastfeeding and/or (2) a positive urine screen for any illicit drug. Importantly, these patients had no clinical signs of alcoholic liver disease, and since the HCV diagnosed patients had the first-time diagnosis, there was no standard of care indication to perform a biopsy (as this study is not a treatment evaluation study). Patients did not show any clinical evidence of liver disease, and overt clinical liver disease was an exclusion criterion in the study; detailed information on eligibility criteria has been published previously [15].

### 2.2. Clinical Data and Laboratory Measures

This study was a one-time study at the admission clinical observational investigation. Demographic information included age, sex, height, weight, and BMI. Blood samples were collected for a comprehensive metabolic panel (CMP) that included a liver panel, fatty acid panel (FAP), and serum trace metals (zinc for this study). Serum Alanine Aminotransferase (ALT), Aspartate Aminotransferase (AST), AST/ALT ratio, Total bilirubin (Tbili), and albumin (ALB) measures were evaluated for the extent of liver injury. Alanine Aminotransferase (ALT) level was used as a reference to assess liver injury (Medline Plus-National Institutes of Health, 2014); 40 IU/L for ALT was used as the upper limit of normal, and values ≥41 IU/L indicated liver injury as per the guideline that existed till 2014. The standard lower limit of normal for serum zinc was 71 mcg/dL in this study. The comprehensive fatty acid panel measured specific and total *ω*3 and *ω*6 FA levels using gas chromatography/mass spectrometry. The screening was performed to identify patients with HCV, which was conducted with COBAS Ampliprep/COBAS Taqman HCV test methodology; HCV diagnosis was a first-time report in Gr. 1 and Gr. 2. Genotyping and RNA quantification for HCV were also performed. No additional proteomic or rapid turnover proteins analyses were in the scope of this study. All ranges and guidelines for testing were assigned and all the study tests were performed by the Department of Laboratory Medicine, NIH, Bethesda, MD, USA.

Drinking history was collected. Heavy drinking is defined as an intake of 15 alcoholic drinks or more per week for males and eight drinks or more per week for females (https://www.cdc.gov/alcohol/faqs.htm). All AUD patients enrolled in this study met the criteria for heavy drinking per the Center for Disease Control recommendation. Timeline followback past 90 days (TLFB90) questionnaire has been a validated and well-established instrument to collect self-reported data on the total number of drinks for each day in the past 90 days [16]. TLFB90 was used in our study to collect drinking history in all the groups. Markers of drinking that were derived from the TLFB90 questionnaire included total drinks in the past 90 days (TD90), the number of drinking days in the past 90 days (NDD90), drinks per drinking day in the past 90 days (DPD90), average drinks per drinking day in the past 90 days (AvgDD90), and heavy drinking days in the past 90 days (HDD90). We also used the lifetime drinking history (LTDH) questionnaire [17] and the number of years as other drinking measures of evaluation in this study (https://pubs.niaaa.nih.gov/publications/AssessingAlcohol/measures.htm). We used the “Controlling Nutritional Status Test” (CONUT) data to establish nutritional status. None of the participants in this study showed signs of overt malnutrition [18].

### 2.3. Statistical Analysis

Differences among demographics, drinking history markers, zinc levels, fatty acid levels, and ALT levels were evaluated with the use of one-way ANOVA for the five groups. Fisher's exact test was used to compare group differences for categorical variables (e.g., sex). Univariate and multivariate linear regression analyses were conducted to analyze the associations between one variable versus one or multiple variables, respectively. Drinking history was used as a covariate where applicable. The significance level was set at *p* < 0.05. The association analysis described the goodness of model fit (adjusted *R*^2^). SPSS 26.0 (IBM, Chicago, IL) and Microsoft Excel 2016 (MS Corp., Redmond, WA) were used for data analyses. Data are presented as mean ± standard deviation (M ± SD) for continuous variables.

## 3. Results

### 3.1. Demographics, Drinking Profile, and HCV Markers

In our study cohort, 25 out of 38 (around 66%) HCV diagnosed patients were comorbid with AUD. There was not much difference in the demographic measures either numerically or statistically in the five study cohorts (Table 1). AUD patients without liver injury (Gr. 3) were borderline overweight and were drinking for the least number of years. HCV + AUD patients showed slightly higher levels of drinking markers compared to the AUD patients (Gr. 3 and Gr. 4). There were only two females in the HCV group; thus, this finding limited the analyses of the sex differences within this cohort (or with other groups). Only two HV subjects reported social drinking in the past 90 days; thus, data were not added/evaluated in Table 1. All AUD patients drank more than 10 drinks per day on average.

There was no significant difference in the HCV RNA quantification (or their converted international unit) values between HCV-only and HCV + AUD groups; however, these levels were higher in the HCV + AUD group (Supplemental Table S1A). Genotype 1A was the most prevalent genetic constitution (52.6%) among the patients diagnosed with HCV in this study (Supplemental Table S1B).

### 3.2. Liver Injury

**Figure 1:**
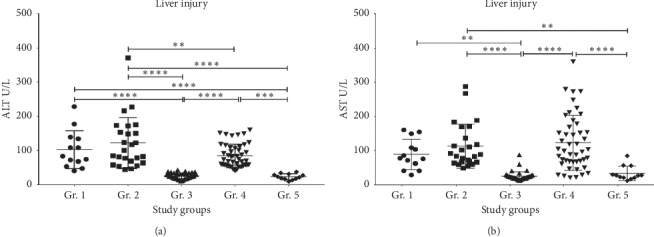
Differences in the liver injury markers in all the study groups. (a) Serum ALT levels in all the study cohorts. (b) Serum AST levels in all the study groups. Data were presented as mean ± standard deviation. Statistical significance was set at *p* ≤ 0.05.

ALT was significantly higher in the HCV + AUD patients (Gr. 2), followed by the HCV-only patients (Gr. 1) and AUD patients with liver injury subsequently (Gr. 4) (Figure 1(a)). HCV + AUD patients showed numerically higher ALT levels than the HCV-only patients; however, this increase was not statistically significant. AUD patients with liver injury showed comparable levels of ALT with HCV-only patients, and the levels between the two groups were not significantly different. AST levels did not show any numerical or statistical difference between Gr.1 and Gr. 2 and Gr. 2 and Gr. 4 (Figure 1(b)). Mean AST was the highest in Gr. 4; however, this could be due to one single patient with AST approaching 400 u/L. Going forward with the analyses, we did not include the healthy volunteer (HV, Gr. 5) data.

### 3.3. Zinc Deficiency and Proinflammatory Shift

**Figure 2:**
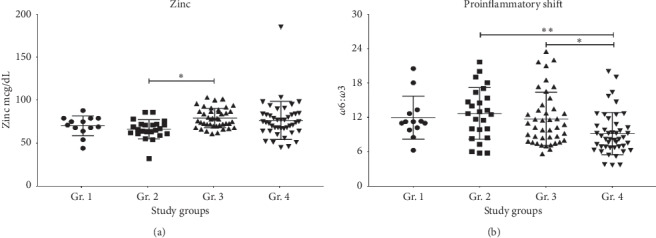
Serum zinc concentrations and plasma assessed proinflammatory shift in all the study groups. (a) Serum zinc level in all the study cohorts. (b) Proinflammatory shift as characterized by *ω*6 : *ω*3 ratio in all the study groups. Data were presented as mean ± standard deviation. Statistical significance was set at *p* ≤ 0.05.

Zinc deficiency and FA dysregulation are already well known in liver disease. The current study focused primarily on the HCV + AUD patients, and data from other cohorts were used for comparison purposes. Zinc deficiency was the most in Gr. 2 (HCV + AUD cohort) followed by HCV-only patients (Gr. 1) (Table 2); however, this lowering in Gr. 2 was not statistically significant compared to Gr. 1 (Figure 2(a)). A similar tendency in the proinflammatory shift was found between Gr. 2 and Gr. 1 response (as characterized by *ω*6 : *ω*3 ratio; Figure 2(b)). *ω*6 : *ω*3 ratio level in Gr. 2 was significantly higher (*p*=0.001) than that in Gr. 4. Noteworthy, Gr. 4 *ω*6 : *ω*3 ratio was lower than Gr. 3, which was consistent with our previous report [19].

### 3.4. Changes in Fatty Acid Spectrum

**Figure 3:**
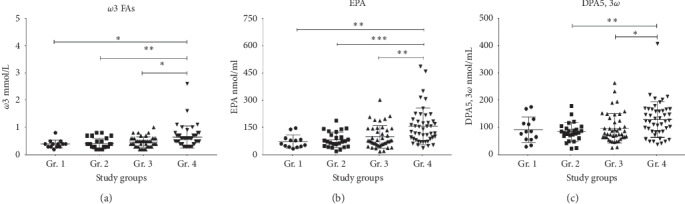
Plasma *ω*3 PUFA measures in HCV-only (Gr. 1), HCV + AUD (Gr. 2), AUD without any liver injury (Gr. 3), and AUD with liver injury (Gr. 4). (a) Total *ω*3 concentration in Gr. 1 through Gr. 4. (b) Plasma eicosapentaenoic acid (EPA) concentration in Gr. 1 through Gr. 4. (c) *ω*3 docosapentaenoic acid (DPA5,3*ω*) concentrations in Gr. 1 through Gr. 4. Data were presented as mean ± standard deviation. Statistical significance was set at *p* ≤ 0.05.

The significantly higher *ω*6 : *ω*3 ratio in the HCV + AUD (Gr. 2) patients discussed above was due to a lowering of the total *ω*3 (Figure 3(a)), which was significantly evident between Gr. 2 and Gr. 4. We did not find these differences in the total *ω*6 values (data not presented). Thus, we evaluated the lowering of FAs in Gr. 2 further. We assessed the total and individual *ω*3 FAs that contributed to the anti-inflammatory response. Both EPA and DPA5,3*ω* were significantly lower in Gr. 2 (HCV + AUD) (Figures 3(b) and 3(c)). DHA values showed only a trend of lowering in the HCV + AUD patients (Gr. 2) compared to the AUD with liver injury patients (Gr. 4) (Supplemental Table S2). Lowering of the concentrations of the candidate *ω*3 PUFAs, specifically, EPA and DPA5,3*ω*, shows greater weightage in the proinflammatory shift more than the increases in the proinflammatory *ω*6 PUFAs. With these findings, we further focused our analyses of the proinflammatory response originating from the lowering of *ω*3 PUFAs on Gr. 2.

### 3.5. Association of PUFAs and Zinc in HCV and AUD Comorbid Patients

**Figure 4:**
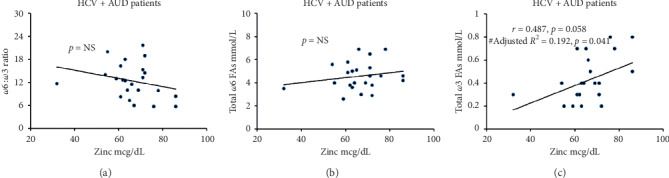
Association of plasma-derived PUFAs involved in inflammation and serum zinc levels in HCV + AUD patients (Gr. 2). (a) Association of proinflammatory shift as characterized by the *ω*6 : *ω*3 ratio and serum zinc levels. (b) Association of total *ω*6 concentrations and serum zinc levels. (c) Association of total *ω*3 concentrations and serum zinc levels. Data analysis showed correlation coefficient: “*r*”; #adjusted analysis with NDD90 as a covariable: *R*^2^. Statistical significance was set at *p* ≤ 0.05. NS: not significant.

Zinc levels did not show any association either with the *ω*6 : *ω*3 ratio (Figure 4(a)) or with the total *ω*6 FAs concentrations (Figure 4(b)). However, there was a trend level of association between the serum zinc level and *ω*3 levels (Figure 4(c)), which augmented to statistical significance when NDD90 was added as a covariable in this regression analysis. These within-group associations were either not significant or showed much lower correlations in Gr. 1 and Gr. 4. With this information, we further evaluated candidate *ω*3 PUFAs that are involved in the anti-inflammatory response in the downstream fashion.

**Figure 5:**
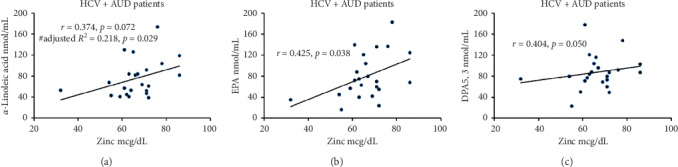
Association of plasma-derived PUFAs involved in inflammation and serum zinc levels in HCV + AUD patients (Gr. 2). (a) Association of *α*-linoleic acid (*α*LA) concentrations and serum zinc levels. (b) Association of eicosapentaenoic acid (EPA) concentrations and serum zinc levels. (c) Association of *ω*3 docosapentaenoic acid (DPA5,3*ω*) concentrations and serum zinc levels. Data analysis showed a correlation coefficient: *r*; #adjusted analysis with NDD90 as a covariable: *R*^2^. Statistical significance was set at *p* ≤ 0.05. NS: not significant.

*α*-Linoleic acid showed a trend level of significant association with lower zinc values in Gr. 2, which augmented when NDD90 was added as a covariable in the same analysis (Figure 5(a)). EPA (Figure 5(b)) and DPA5,3*ω* (Figure 5(c)) showed a significant association with the serum zinc concentrations independently. The association of zinc deficiency showed a close association with the candidate *ω*3 PUFAs in Gr. 2. HCV RNA quantification was significantly associated with DPA5,3*ω* and added covariable NDD90 (*p*=0.035) in HCV + AUD patients, although in HCV patients it was only trending (data not plotted).

## 4. Discussion

Approximately two-thirds of the HCV diagnosed patients exhibited heavy alcohol drinking. Heavy drinking HCV patients showed higher liver injury [20] as characterized by the serum ALT levels. Notably, these patients did not show any outwardly different drinking patterns than the AUD patients who had a liver injury. HCV patients who did not drink also had clinically relevant levels of ALT; however, they were comparatively much lower than the ALT levels in HCV-infected AUD patients. Serum zinc levels in the HCV-infected heavy drinkers were the lowest, consistent with a higher proinflammatory shift. On the other hand, we found a lowered proinflammatory shift in AUD patients with liver injury (Gr. 4), which is consistent with our previous study [19].

Several polyunsaturated fatty acids (PUFAs) including EPA and DHA inhibit HCV replication *in vitro* using the HCV RNA replicon system considerably [21]. In our study, we found a generalized drop in the total *ω*3, EPA, and DPA5,3*ω* and DHA PUFAs in both the HCV-only and HCV + AUD groups. Previously, *ω*3 supplementation has shown efficacy in the medical management of HCV patients who priorly were nonresponders to the combination antiviral therapy [22]. We found that the HCV + AUD patients also had higher HCV RNA quantification, which was significantly predictable by the DPA5,3*ω* level with respect to heavy drinking. Anti-HCV activities of the selective PUFAs have been reported previously [23]. Our study showed that, in HCV + AUD patients, elevated RNA quantification was shown compared to the HCV-only patients.

Zinc depletion has been abundantly reported with liver disease [24]; importantly, it is lower in the HCV diagnosed patients [25]. Zinc levels were also deficient in AUD patients who exhibited early-stage ALD [15]. In one report, altered PUFAs were observed in HIV-HCV-infected heavy drinkers [26]. In our study, findings on zinc deficiency were consistent in HCV-infected patients, both those who had only HCV infection alone and with AUD diagnosis, and those who also drank heavily with or without liver injury. However, this deficiency in zinc was most severe in HCV + AUD patients. Zinc metabolism in ALD gets altered [27], and zinc deficiency [28] causes major dysregulation in several pathological pathways involved in the initiation and progression of ALD [29]. A therapeutic role for zinc supplementation has shown great promise in ALD, especially in alcoholic cirrhosis and alcoholic hepatitis [30, 31].

Zinc has a pivotal role in the hepatic regulation of fatty acids; it aids in the downstream process of anti-inflammatory PUFAs [32, 33]. Therefore, a low zinc level likely lowered the downstream synthesis of omega-3 PUFAs systematically in HCV + AUD patients (Supplemental Table S2). Previously, we have reported that AUD heavy drinkers with HIV comorbidity showed a close association of proinflammatory *ω*6 PUFA and low zinc [19]. In this study, we found that low zinc was closely associated with total *ω*3 PUFAs and EPA and DPA5,3*ω* [34] in HCV-infected heavy drinkers. One recent review has described the role of zinc as a cofactor in the modulation of desaturase activity involved in FA absorption, oxidation, metabolism, and incorporation [12]. The importance of our study was that low zinc and candidate *ω*3 PUFAs were significantly associated with HCV + AUD patients only. The interaction of hypozincemia and the lowering of anti-inflammatory FAs in HCV-infected heavy drinkers likely played a pathological role in the proinflammatory shift.

NDD90 as a heavy drinking marker showed a close association with candidate *ω*3 fatty acids, especially DPA5,3*ω*, in the expression of HCV RNA quantification. Its role was also significant in the association analyses for deficient *ω*3 PUFAs and low zinc. The connection of specific markers of heavy drinking with the exacerbation of metabolic dysregulation in liver injury has been recently highlighted [35]. The number of drinking days past 90 days (NDD90) was a significant marker that was observed in our study. It seems that how many days an individual drinks heavily (NDD90) in an assessment period is consequential in the changes in the anti-inflammatory *ω*3 PUFAs in HCV-infected heavy drinkers, which was a new finding in our study.

Our study has several limitations. We had smaller number of females in each group; however, in HCV-infected groups, this difference was numerically significant. This study was a single time-point clinical observational investigation on the characterization of low zinc and omega-3 alterations in a unique HCV and AUD comorbid cohort. Thus, neither longitudinal interpretations or treatment outcomes of zinc or omega-3 supplementation were not evaluated. The plasma or tissue levels of PGs, LTs, TXs, and lipoxins were not measured, which could have further identified specific role of the product-associated proinflammatory response, synthesized during the downstream process of PUFA. There was no biopsy performed on the study patients since this was an observational study only. This study did not use a mechanistic approach, which was beyond the scope of this study design. Dietary FAs were not studied in this investigation; however, none of the patients showed any malnutrition at intake (Table 2). We did not study the type of alcohol [36]; our study focused on the patterns and amount of alcohol intake as per the guideline of NIAAA as described in the methods section.

Lowering of the concentrations in *ω*3 PUFAs, DPA5,3*ω* and EPA, likely plays a significant role in the proinflammatory shift in HCV-infected AUD patients. Serum zinc lowered to a clinically deficient level in the same cohort. This deficiency was a strong predictor of the lowering of total and candidate *ω*3 PUFAs, exclusively, in the HCV + AUD patients. The number of drinking days (NDD90) could be a good marker in HCV-infected heavy drinkers and a key to identify the changes that can incur in the *ω*3 PUFAs in such comorbid cohort. This report extends the need to elucidate the anti-HCV mechanism in comorbid AUD patients by a combination of specific anti-inflammatory PUFAs [37] and zinc supplementation [38] that may lead to the development of novel molecular-based therapeutic agents and direct therapeutic mechanistic studies in this special cohort.

## 5. Conclusions

Depletion in the concentrations of *ω*3 PUFAs, DPA5,3*ω* and EPA, is involved in the proinflammatory shift in HCV diagnosed individuals, especially comorbid with AUD diagnosis. Clinically deficient zinc concentrations are observed in HCV patients, with further lowering in HCV-infected AUD patients. This deficiency in both *ω*3 PUFAs and zinc concentrations is highly correlated. The assessment of the number of drinking days (NDD90) could be a good marker in HCV-infected heavy drinkers to identify the changes that are involved in the proinflammatory response.

## Figures and Tables

**Table 1:** Demographic and drinking profile of HCV patients without or with AUD, AUD patients (without HCV) without or with liver injury, and healthy volunteers.

Groups/measures	HCV patients		AUD		Healthy volunteers (*n* = 12) (Gr. 5)
	HCV-only (Gr. 1) (*n* = 13)	HCV + AUD (Gr. 2) (*n* = 25)	AUD without liver injury (Gr. 3) (*n* = 42)	AUD with liver injury (Gr. 4) (*n* = 46)	

Demographics					
Sex	Male = 11 Female = 2	Male = 18 Female = 7	Male = 28 Female = 14	Male = 34 Female = 12	Male = 5 Female = 7

Age (years)	47.83 ± 7.7	47.25 ± 5.8	39.33 ± 10.83	43.76 ± 10.0	40.98 ± 11.7
Height (cm)	173.25 ± 9.9	176.60 ± 9.11	170.81 ± 9.97	173.65 ± 7.6	169.00 ± 10.9
Weight (kg)	73.97 ± 13.11	78.65 ± 12.39	79.23 ± 19.46	77.46 ± 12.4	68.06 ± 13.2
BMI	24.65 ± 3.57	25.22 ± 3.87	26.49 ± 4.7	25.66 ± 3.5	23.58 ± 5.3

Drinking profile					
LTDH (years)	17.60 ± 11.06	16.86 ± 11.5	13.10 ± 8.4	15.11 ± 10.2	NA
TD90	159.25 ± 46.7	1118.36 ± 443.08	841.81 ± 458.1	1052.57 ± 442.7	NA
AvgDPDD90	5.50 ± 1.9	14.52 ± 5.08	12.38 ± 6.0	13.85 ± 5.1	NA
NDD90	33.5 ± 19.3	78.24 ± 18.1	67.57 ± 21.9	76.46 ± 16.9	NA
HDD90	20.00 ± 16.5	76.04 ± 18.45	62.69 ± 21.6	73.43 ± 19.5	NA

BMI: body mass index, LTDH: lifetime drinking history, TD90: total drinks in the past 90 days, AvgDPDD90: average drinks per drinking day in the past 90 days, NDD90: number of drinking days in the past 90 days, HDD90: heavy drinking days in the past 90 days. Data were represented as mean ± SD and statistical significance was set at *p* < 0.05. NA: not applicable.

**Table 2:** Liver injury, nutritional status, and serum nutrients in the study cohorts.

Groups/measures	HCV patients		AUD		Healthy volunteers (Gr. 5)
	HCV-only (Gr. 1)	HCV + AUD (Gr. 2)	AUD without liver injury (Gr. 3)	AUD with liver injury (Gr. 4)	

Liver panel					
ALT (U/L)	102.62 ± 54.85	121.68 ± 74.26	26.40 ± 8.44	84.48 ± 33.55	23.92 ± 8.65
AST (U/L)	88.85 ± 43.82	112.84 ± 64.41	25.02 ± 12.99	122.65 ± 80.30	33.67 ± 21.1
AST : ALT ratio	0.89 ± 0.24	1.03 ± 0.42	1.01 ± 0.48	1.44 ± 0.83	1.39 ± 0.53
Tbili (*μ*mol/L)	0.68 ± 0.23	0.71 ± 0.39	0.69 ± 0.61	0.72 ± 0.61	0.73 ± 0.70
ALB (g/dL)	4.02 ± 0.36	3.83 ± 0.32	4.10 ± 0.32	4.10 ± 0.46	4.05 ± 0.32

Serum zinc and CONUT					
Zn (mcg/dL)	70.23 ± 11.53	63.50 ± 17.40	79.20 ± 11.25	72.89 ± 26.80	72.10 ± 15.73
CONUT score	1.17 ± 0.94	1.78 ± 1.28	0.85 ± 1.13	1.23 ± 1.55	1.10 ± 1.31

*ω*3 and *ω*6 polyunsaturated fatty acids participating in inflammation in AUD and HCV + AUD patients. ALT: alanine transaminase; AST: aspartate transaminase; Tbili: Total bilirubin; ALB: albumin; Zn: serum zinc; CONUT: Controlling Nutritional Status.

## Data Availability

Data will be provided on request based on reasonable queries only.

## Abbreviations

AUD: Alcohol use disorder
ALD: Alcoholic liver disease
ALB: Albumin
ALP: Alkaline phosphatase
ALT: alanine aminotransferase
AST: Aspartate aminotransferase
AST: ALT: AST by ALT ratio
CS: Clinically significant
DHA: Docosahexaenoic acid
DPA 5,3*ω*: Docosapentaenoic acid
FA: Fatty acids
Gr. 1: HCV-only
Gr. 2: HCV + AUD
Gr. 3: AUD without liver injury
Gr. 3: AUD with liver injury
Gr. 5: HV
HCV: Hepatitis C virus
HCV + AUD: Patients with HCV and AUD comorbidity
HV: Healthy volunteers
PUFAs: Polyunsaturated fatty acids
Tbili: Total bilirubin
TLFB90: Timeline followback past 90 days
TD90: Total drinks in the past 90 days
AvgD90: Average drinks in the past 90 days
HDD90: Heavy drinking days in the past 90 days
NDD90: Number of drinking days in the past 90 days
*ω*3-EPA: Eicosapentaenoic acid
*ω*6 : *ω*3: Omega 6 by omega 3 ratio.

## Supplementary Materials

Supplementary Materials Supplemental Table S1: (A) Diagnostic biomarkers of Hep C infection. (B) Infection genotypes as reported together for the study groups 1 (HCV-only) and 2 (HCV + AUD). Supplemental Table S2: *ω*3 and *ω*6 polyunsaturated fatty acids participating in the inflammation in HCV, AUD, and HCV + AUD patients.

## References

[1] Davis G., Albright J. E., Cook S. F., Rosenberg D. M. (2003). Projecting future complications of chronic hepatitis C in the United States. *Liver Transplantation*.

[2] Ivanov A., Bartosch B., Smirnova O., Isaguliants M., Kochetkov S. (2013). HCV and oxidative stress in the liver. *Viruses*.

[3] Rehm J., Dawson D., Frick U. (2014). Burden of disease associated with alcohol use disorders in the United States. *Alcoholism: Clinical and Experimental Research*.

[4] Bataller R., Arteel G. E., Moreno C., Shah V. (2019). Alcohol-related liver disease: time for action. *Journal of Hepatology*.

[5] Thursz M., Szabo G., Kamath P. S. (2019). Alcohol-related liver disease: areas of consensus, unmet needs and opportunities for further study. *Journal of Hepatology*.

[6] Novo-Veleiro I. (2016). Alcoholic liver disease and hepatitis C virus infection. *World Journal of Gastroenterology*.

[7] Zirnheld K. H., Warner D. R., Warner J. B., Hardesty J. E., McClain C. J., Kirpich I. A. (2019). Dietary fatty acids and bioactive fatty acid metabolites in alcoholic liver disease. *Liver Research*.

[8] Read S. A., Obeid S., Ahlenstiel C., Ahlenstiel G. (2019). The role of zinc in antiviral immunity. *Advances in Nutrition*.

[9] Suda T., Okawa O., Shirahashi R., Tokutomi N., Tamano M. (2019). Changes in serum zinc levels in hepatitis C patients before and after treatment with direct‐acting antiviral agents. *Hepatology Research*.

[10] Vatsalya V., Cave M. C., Kumar R. (2019). Alterations in serum zinc and polyunsaturated fatty acid concentrations in treatment-naive HIV-diagnosed alcohol-dependent subjects with liver injury. *AIDS Research and Human Retroviruses*.

[11] Barocas J. A., Armah K. S., Cheng D. M. (2019). Zinc deficiency and advanced liver fibrosis among HIV and hepatitis C co-infected anti-retroviral naïve persons with alcohol use in Russia. *PloS One*.

[12] Tsoukalas D., Alegakis A. K, Fragkiadaki P (2019). Application of metabolomics part II: focus on fatty acids and their metabolites in healthy adults. *International Journal of Molecular Medicine*.

[13] Shoreibah M., Anand B. S., Singal A. K. (2014). Alcoholic hepatitis and concomitant hepatitis C virus infection. *World Journal of Gastroenterology*.

[14] Sobell L. C., Sobell M. B. (1992). Timeline follow-back. *Measuring Alcohol Consumption*.

[15] Vatsalya V., Kong M., Cave M. C. (2018). Association of serum zinc with markers of liver injury in very heavy drinking alcohol-dependent patients. *The Journal of Nutritional Biochemistry*.

[16] Sobell L. C., Sobell M. B., Connors G. J., Agrawal S. (2003). Assessing drinking outcomes in alcohol treatment efficacy studies: selecting a yardstick of success. *Alcoholism: Clinical & Experimental Research*.

[17] Skinner H. A., Sheu W. J. (1982). Reliability of alcohol use indices. The lifetime drinking history and the MAST. *Journal of Studies on Alcohol*.

[18] Fukushima K., Ueno Y., Kawagishi N. (2011). The nutritional index “CONUT” is useful for predicting long-term prognosis of patients with end-stage liver diseases. *The Tohoku Journal of Experimental Medicine*.

[19] Vatsalya V., Song M., Schwandt M. L. (2016). Effects of sex, drinking history, and omega-3 and omega-6 fatty acids dysregulation on the onset of liver injury in very heavy drinking alcohol-dependent patients. *Alcoholism: Clinical and Experimental Research*.

[20] Wiley T. E., McCarthy M., Breidi L., McCarthy M., Layden T. J. (1998). Impact of alcohol on the histological and clinical progression of hepatitis C infection. *Hepatology*.

[21] Kapadia S. B., Chisari F. V. (2005). Hepatitis C virus RNA replication is regulated by host geranylgeranylation and fatty acids. *Proceedings of the National Academy of Sciences*.

[22] Sheridan D. A., Bridge S. H., Crossey M. M. E. (2014). Omega-3 fatty acids and/or fluvastatin in hepatitis C prior non-responders to combination antiviral therapy - a pilot randomised clinical trial. *Liver International*.

[23] Leu G.-Z., Lin T.-Y., Hsu J. T. A. (2004). Anti-HCV activities of selective polyunsaturated fatty acids. *Biochemical and Biophysical Research Communications*.

[24] Mohammad M. K., Zhou Z., Cave M., Barve A., McClain C. J. (2012). Zinc and liver disease. *Nutrition in Clinical Practice*.

[25] Moriyama M., Matsumura H., Fukushima A. (2006). Clinical significance of evaluation of serum zinc concentrations in C-viral chronic liver disease. *Digestive Diseases and Sciences*.

[26] Vatsalya V., Barve S. S., McClain C. J., Ramchandani V. A. (2016). Elevated linoleic acid (a pro-inflammatory PUFA) and liver injury in a treatment naive HIV-HCV co-infected alcohol dependent patient. *Journal of Biosciences and Medicines*.

[27] Sullivan J. F., Heaney R. P. (1970). Zinc metabolism in alcoholic liver disease. *The American Journal of Clinical Nutrition*.

[28] Kiilerich S., Dietrichson O., Loud F. B. (1980). Zinc depletion in alcoholic liver diseases. *Scandinavian Journal of Gastroenterology*.

[29] McClain C. J., Antonow D. R., Cohen D. A., Shedlofsky S. I. (1986). Zinc metabolism in alcoholic liver disease. *Alcoholism: Clinical and Experimental Research*.

[30] McClain C., Vatsalya V., Cave M. (2017). Role of zinc in the development/progression of alcoholic liver disease. *Current Treatment Options in Gastroenterology*.

[31] Zhou Z., Wang L., Song Z., Saari J. T., McClain C. J., Kang Y. J. (2005). Zinc supplementation prevents alcoholic liver injury in mice through attenuation of oxidative stress. *The American Journal of Pathology*.

[32] Ayala S., Brenner R. R. (1983). Essential fatty acid status in zinc deficiency. Effect on lipid and fatty acid composition, desaturation activity and structure of microsomal membranes of rat liver and testes. *Acta Physiologica Latino Americana*.

[33] Cunnane S. (1988). Role of zinc in lipid and fatty acid metabolism and in membranes. *Progress in Food & Nutrition Science*.

[34] Bettger W. J., Reeves P. G., Moscatelli E. A., Reynolds G., O’Dell B. L. (1979). Interaction of zinc and essential fatty acids in the rat. *The Journal of Nutrition*.

[35] Vatsalya V., Hassan H. Z., Kong M. (2019). Exacerbation of hangover symptomology significantly corresponds with heavy and chronic alcohol drinking: a pilot study. *Journal of Clinical Medicine*.

[36] Bellentani S., Saccoccio G., Costa G. (1997). Drinking habits as cofactors of risk for alcohol induced liver damage. *Gut*.

[37] Bassendine M. F., Sheridan D. A., Felmlee D. J., Bridge S. H., Toms G. L., Neely R. D. G. (2011). HCV and the hepatic lipid pathway as a potential treatment target. *Journal of Hepatology*.

[38] Takagi H., Nagamine T., Abe T. (2001). Zinc supplementation enhances the response to interferon therapy in patients with chronic hepatitis C. *Journal of Viral Hepatitis*.

