# Supplementary material for: Dysregulation in Plasma ω3 Fatty Acids Concentration and Serum Zinc in Heavy Alcohol-Drinking HCV Patients
Source: Adv Virol. 2020 Jun 9;2020:7835875. doi: 10.1155/2020/7835875 (PMC7301182; doi:10.1155/2020/7835875)
Supplement: Supplementary Materials — Supplemental Table S1: (A) Diagnostic biomarkers of Hep C infection. (B) Infection genotypes as reported together for the study groups 1 (HCV-only) and 2 (HCV + AUD). Supplemental Table S2: ω3 and ω6 polyunsaturated fatty acids participating in the inflammation in HCV, AUD, and HCV + AUD patients. [file 7835875.f1.pdf]

Supplementary Online Material

Title: Dysregulation in Plasma ω-3 Fatty Acids Concentration and Serum Zinc in Heavy Alcohol-Drinking HCV Patients

Lead Author: Vatsalya Vatsalya

Supplemental Tables

Supplemental Table S1: A. Diagnostic biomarkers of Hep C infection. B. Infection genotypes as reported together for the study groups 1 (HCV only) and 2 (HCV+AUD).

A

Measures	Drinking Groups	Mean	Std. Deviation	N	p-value
HCV RNA Quan (Copies/mL)	No AUD	6903929.08	8113935.421	13	NS
	AUD	8755668.00	7079236.383	25	
	Total	8122178.37	7392713.575	38	
HCV RNA (IU/ML)	No AUD	2557010.77	3005161.267	13	NS
	AUD	3242840.00	2621939.401	25	
	Total	3008214.21	2738042.065	38	

B

Types	Frequency	Valid Percent	Cumulative %
No Genotyping	3	7.9	7.9
1	5	13.2	21.1
1A	20	52.6	73.7
1B	6	15.8	89.5
3A	3	7.9	97.4
AB	1	2.6	100.0
Total	38	100.0	

Supplemental Table S2: ω-3 and ω-6 polyunsaturated fatty acids participating in inflammation in HCV, AUD and HCV+AUD patients.

Groups/ Measures	HCV Patients		AUD		Normal Range (nmol/mL)
	HCV only (Gr. 1)	HCV + AUD (Gr. 2)	AUD without liver injury (Gr. 3)	AUD with liver injury (Gr. 4)	
Candidate Omega – 6 PUFAs (Pro-inflammatory)					
Linoleic acid	3080.23±439.39	3214.96±800.98	3388.36±743.16	3419.89±838.42	2270 – 3850
γ-Linolenic acid	82.15±43.39	73.80±34.39	81.02±32.95	94.62±42.39	16 – 150
DGLA	117.54±34.96	119.92±49.88	115.79±35.87	136.60±49.49	50 – 250
Arachidonic acid	1051.54±272.05	1128.32±347.96	1208.43±460.85	1259.40±442.98	520 – 1490
DTA	25.92±10.76	25.56±11.16	37.26±22.56	38.91±28.35	10 – 80
DPA5 6ω	40.00±24.85	35.84±16.85	37.26±22.56	38.91±28.35	10 – 70
Candidate Omega – 3 PUFAs (Anti-inflammatory)					
αLA	82.15±43.39	73.80±34.39	81.02±32.95	94.62±42.39	50 - 130
EPA	69.15	78.60±42.01	94.64±61.26	152.29±100.56	14 - 100
DPA5 3ω	91.23±46.24	83.16±34.66	96.90±53.20	128.69±65.47	20 - 210
DHA	154.85±74.80	186.72±110.20	196.83±96.96	269.64±224.78	30 - 250
ω6:ω3 ratio	11.93±3.73	12.66±4.49	11.72±4.64	9.15±3.70	10 – 15 (no units)

PUFAs: Polyunsaturated fatty acids, ω3 – αLA: Alpha linoleic Acid; EPA: Eicosapentaenoic Acid, DPA 5 3ω: Docosapentaenoic Acid, DHA: Docosahexaenoic acid, . ω6 - DGLA: Dihomo-γ-Linolenic acid, DTA: Docosatetraenoic acid, DPA5 6ω: Docosapentaenoic Acid. Unit for PUFAs: nmol/mL. Data represented as Mean±SD. H: Clinically High.
